# Gastroprotective Activity of *Parastrephia quadrangularis* (Meyen), Cabrera from the Atacama Desert

**DOI:** 10.3390/molecules23092361

**Published:** 2018-09-15

**Authors:** Alejandro Ardiles, Ruth Barrientos, Mario J. Simirgiotis, Jorge Bórquez, Beatriz Sepúlveda, Carlos Areche

**Affiliations:** 1 Departamento de Química y Farmacia, Facultad de Ciencias, Universidad Católica del Norte, Angamos 0610, Antofagasta 1240000, Chile; alejandro.ardiles@ucn.cl; 2 Instituto de Farmacia, Facultad de Ciencias, Universidad Austral de Chile, Casilla 567, Valdivia 5090000, Chile; ruth.barrientos@alumnos.uach.cl; 3 Center for Interdisciplinary Studies on the Nervous System (CISNe), Universidad Austral de Chile, Valdivia 5090000, Chile; 4 Laboratorio de Productos Naturales, Departamento de Química, Facultad de Ciencias Básicas, Universidad de Antofagasta, Casilla 170, Antofagasta 1240000, Chile; Jorge.borquez@uantof.cl; 5 Departamento de Ciencias Químicas, Universidad Andres Bello, Campus Viña del Mar, Quillota 980, Viña del Mar 2520000, Chile; bsepulveda@uc.cl; 6 Departamento de Química, Facultad de Ciencias, Universidad de Chile, Santiago 8320198, Chile

**Keywords:** clerodanes, tremetones, gastroprotective, LC-MS/MS, gastric ulcer, endemic plants

## Abstract

Forty-three metabolites including several methoxylated flavonoids, tremetones, and *ent*-clerodane diterpenes were accurately identified for the first time in the ethanolic extract of *P. quadrangularis* by means of hyphenated UHPLC-quadrupole Orbitrap mass spectrometry, and seven isolated compounds were tested regarding gastroprotective activity using the HCl/EtOH-induced lesion model in mice. A new tremetone (compound **6**) is reported based on spectroscopic evidence. The isolated clerodanes and tremetones showed gastroprotective activity in a mouse model, evidenced by compound **7** (*p*-coumaroyloxytremetone), which showed the highest gastroprotective activity (76%), which was higher than the control drug lansoprazole (72%). Our findings revealed that several constituents of this plant have gastroprotective activity, and particularly, *p*-coumaroyloxytremetone could be considered as a lead molecule to explore new gastroprotective agents. This plant is a rich source of biologically active tremetones and terpenoids which can support the ethnobotanical use of the plant.

## 1. Introduction

**Figure 1:**
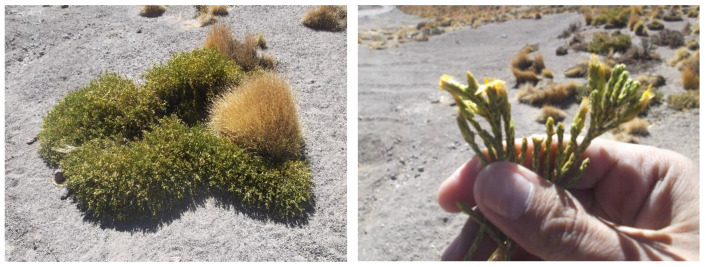
Photographs of aerial parts of *Parastrephia quadrangularis* collected in El tatio, Atacama Desert, in November 2015.

*Parastrephia quadrangularis* (Meyen) Cabrera, (Figure 1) commonly known as *Tola-Tola* (Alpachtola or Burru suputula, Aymara names) since pre-Hispanic times, is a resinous shrub that grows up to 2 m high, typically found in dry semi-arid places in the Cordillera of the Central Andes in the *Puna* habitats, at altitudes of 3500 to 5000 m above the sea level. This plant is medicinal and used for gastrointestinal ailments, plus the treatment of urinary and respiratory diseases, fever, altitude sickness, and to treat bone dislocations and bruises [1,2,3,4], besides cattle feeding in the Atacama Desert [5]. Furthermore, *Parastrephia* (tola) is an important highland genus of South American perennial plants in the aster (sunflower) family (Asteraceae) growing in the Altiplano of Chile, Bolivia, Argentina, and Peru. Many interesting bioactivities were reported for plants within this genus. For example, the plant *Parastrephia lucida* (Meyen) Cabrera is used in traditional medicine as an antiseptic and anti-inflammatory [6]. The plant resin is also used for the healing of wounds and showed inhibition of arachidonic acid metabolism [7]. The related plant *P. lepidophylla* showed antifungal activity on some phytopatogenic fungi of lemon [8] and inhibition of cell proliferation using Caco-2 cells [9]. In addition, the infusions of *P. lepidophylla and P. lucida* showed a protective effect agaist oxidative damage on human erythrocytes [10]. From these genera, antioxidant and analgesic tremetone (5-acetyl-2,3-dihydro-2-isopropenyl-benzofuran) derivatives were isolated [11,12]. Moreover, bioactive constituents of snakeroot (*Eupatorium rugosum*) and several rayless goldenrods (especially *Haplopappus heterophyllus*) and other species are reported to be tremetones, causing milk sickness in humans and trembles in livestock [13,14]. 

Regarding the phytochemical components of *P. quadrangularis*, a poly-methylated flavonoid: 5,7- dihydroxy-3,8,3′,4′-tetramethoxyflavone, some common coumarins and tremetones were reported [12,15]. Recently, other tentative molecules (coumaroyloxytremetone-*O*-hexoside and coumaroyloxytremetone *C*-hexoside) found in a sample from Argentina were suggested using only low-resolution mass spectrometry [16]. Some extracts of this species showed antifungal properties against Fusarium verticilloides which were attributed to the presence of tremetones in the active fractions of the plant [16]. On the other hand, medicinal tinctures are extracts of the active metabolites of the most usable part of a medicinal plant; For this, an extraction method is used in which the plants are submerged or macerated for days in mixtures of edible ethanol water or edible pure ethanol [17,18]. Moreover, one out of five persons suffer from ulcers associated to diet, stress, and certain drugs, due to an imbalance among aggressive factors (bile and hydrochloride acids, pepsins, hypoxia, drugs, and alcohol) and defensive factors (mucose blood flow, nitric oxide, sulfhydryl, growth factors bicarbonate, prostaglandins and mucus) in the stomach. Medicines used in the treatment of gastric ulcers are mainly H2-receptor antagonists, anti-acids, and proton-pump inhibitors and when the gastric ulcer is produced by *Helicobacter pylori*, antibiotics are included in the treatment. In this regard, numerous pharmacological agents with known anti-ulcer activity produce severe collateral effects, showing the need for new agents, including natural products which can be valuable as antiulcer agents [19,20,21]. 

Following our program to analyze and isolate interesting bioactive compounds from the Atacama Desert flora [22,23,24], we report in this study the gastroprotective activity of several compounds isolated from this plant; furthermore, the high resolution UHPLC fingerprinting analysis of the ethanolic extract (medicinal tincture) of this important Aymara plant is reported for the first time. 

## 2. Results and Discussion

### 2.1. Isolation and Identification of the Compounds in Parastrephia Quadrangularis Extract

**Figure 2:**
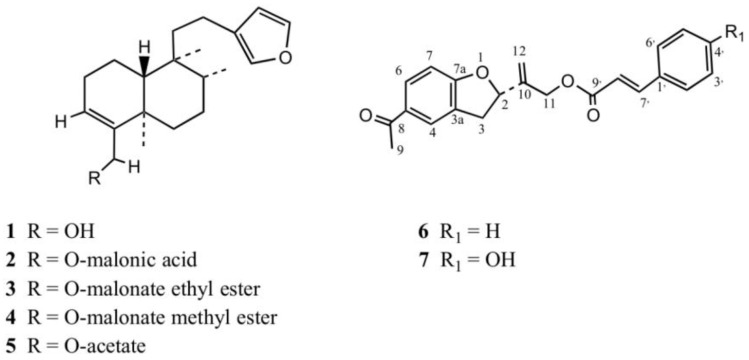
Structures of clerodanes (**1**–**5**) and tremetones (**6**–**7**) isolated from *P. quadrangularis*.

Several isolation steps of the ethanol extract of *P. quadrangularis* allowed the isolation of the known clerodane diterpenes (Figure 2): **1** (bacchalineol), **2** bacchalineol 18-*O*-malonic acid) **3** (bacchalineol 18-*O*-malonate methyl ester), **4** (bacchalineol 18-*O*-malonate ethyl ester), and **5** (bacchalineol 18-*O*-acetate) [25]. In addition, the new tremetone **6** plus the known tremetone **7** [26] were isolated, together with the known methylated flavones 5,7-dihydroxy-3,8,3′,4′-tetramethoxyflavone [15], 3′,4′-dimethoxymyricetin [27], 3,7,3′-trimethoxyquercetin, and hesperetin plus the coumarins umbelliferone and scopoletin [28].

After data comparison with other tremetones [12], and examination of the NMR spectra we realized that compound **6** (Figure 2) showed similar NMR data to that reported for compound **7** [26], particularly the missing of a doublet of doublets in the proton spectra corresponding to the aromatic ring of the coumaroyl moiety (Table S1 and Figure S2, Supplementary Material) plus similar IR bands, but differences in the substitution pattern of only the aromatic protons of the cynnamoyl attached to the tremetone, in the ^1^H- and ^13^C-NMR spectra evidenced by 1-D and 2-D NMR analyses (HMBC, HMQC, Tables S1 and S2, Supplementary Material), which led to the identification of **6** as a *p*-cinnamoyloxyltremetone, a newly reported compound.

**Figure 3:**
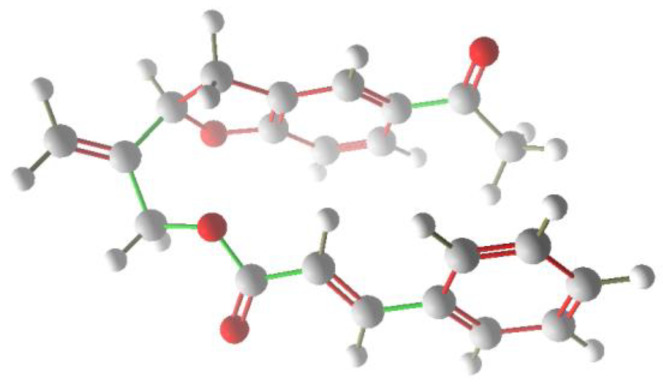
Minimized molecule of compound **6** (Gaussian 9.0, MM1).

*p*-Cinnamoyloxytremetone (compound **6**, Figure 3) IR (CCl_4_, neat) 1650 cm^−1^ (CO), 1685 (PhCO), 1610, 1590 (Ph). ${\left[\alpha \right]}_{D}^{25}$ = −0.4, c = 2.2. Proton NMR (^1^H Bruker Avance 400 MHz, CDCl_3_) δ ppm: 7.85 (1H, br s, H-4), 7.83 (1H, br d, *J* = 8.5 Hz, H-6), 7.65 (1H, d, *J* = 15.9 Hz, H-7′), 7.53 (2H, d, *J* = 8.6 Hz, H-3′, H-5′), 7.40 (2H, d, *J* = 8.6 Hz, H-2′, H-6′), 7.40 (1H, m, H-4′), 6.84 (1H, d, *J* = 8.3 Hz, H-7), 6.39 (1H, d, *J* = 15.9 Hz, H-8′), 5.46 (1H, dd, *J* = 8.8, 8.6 Hz, H-2), 5.38 (2H, d, *J* = 8.8 Hz, H-12), 4.84 (2H, q, *J* = 13.4 Hz, H-11a-H-11b), 3.50 (1H, dd, *J* = 15.9, 9.8 Hz, H-3β), 3.28 (1H, dd, *J* = 15.9, 7.8 Hz, H-3α), 2.50 (3H, s, COCH_3_). ^13^C-NMR (CDCl_3_) δ ppm: 196.8 (C-8), 166.9 (C-9′), 162.8 (C-7a), 125.5 (C-4), 145.5 (C-7′), 141.8 (C-10), 130.6 (C-6), 134.4 (C-5), 129.0 (C-3′), 129.0 (C-5′), 129.0 (C-3a), 128.5 (C-4′), 127.2 (C-1′), 128.2 (C-2′), 128.2 (C-6′), 114.8 (C-12), 109.4 (C-7), 117.8 (C-8′), 84.6 (C-2), 63.8 (C-11), 35.1 (C-3), 26.4 (C-9). HR-ESI-MS spectra: See Table 1.

### 2.2. Full Metabolome Identification by UHPLC-PDA-MS 

**Figure 4:**
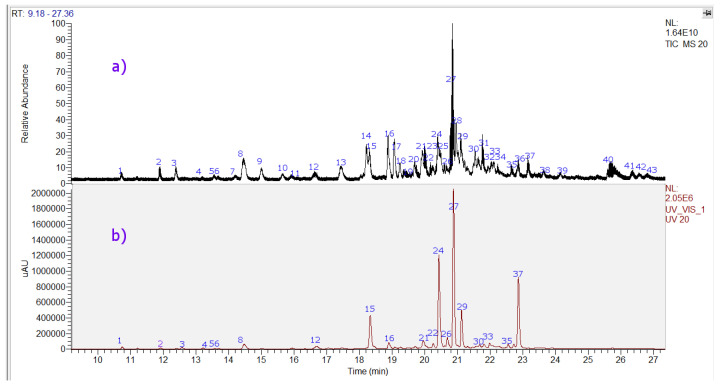
UHPLC Chromatograms of *Parastrephia quadrangularis* resin extract. (**a**) TIC total ion current, negative mode (**b**) UV at 280 nm.

Forty-three compounds were identified including two tremetones (peaks 27 and 37, the last one a new compound), two phenolic acids (peaks 1 and 3), eighteen flavonoids (peaks 4–16, 20, 21, 26, 38, and 41), and twelve diterpenoids, (peaks 17, 18, 22, 23, 25, 28, 29, 31, 32, 34, 36, and 39 in the chromatogram of the ethanol extract of *P. quadrangularis.* (Figure 4, Table 1). Figure S1a–n (Supplementary Materials) show spectra and structures of compounds detected as examples. The detailed identification is explained below.

#### 2.2.1. Phenolic Acids

Peak 1 was identified as caffeoyl-quinic acid (DiCOA, C_25_H_23_O_12_^−^, Figure S1a) [23] and peak 3 with a [M − H]^-^ ion at *m*/*z*: 179.0344 was identified as caffeic acid (C_9_H_7_O_4_^−^).

#### 2.2.2. Coumarins

Peaks 42 and 43 were identified as umbelliferone and scopoletin by spiking experiments with authentic standards. Peak 2 with a pseudomolecular ion at *m*/*z* 353.02919 was identified as the dicoumarin euphorbetin (Figure S1b) [29].

#### 2.2.3. Flavonols

Peak 7 with a pseudomolecular ion at *m*/*z* 315.05090 (C_16_H_11_O_7_^−^) was identified as isorhamnetin, [23] identity confirmed by using co-injection with an authentic standard, and peak 6 as the flavonol kaempferol (C_15_H_9_O_6_^−^), respectively. The 3,7-methoxilated derivatives of isorhamnetin, peaks 14 and 24 (7-*O*-methyl-isorhamnetin and 3,7-di-*O*-methyl-isorhamnetin) were also detected. Peaks 8, 11, and 12 with molecular ions at *m*/*z* 375.07202, 345.06152 and 345.06149 were identified as methoxylated myricetin derivatives with molecular formulas C_18_H_15_O_9_^−^, C_17_H_13_O_8_^−^, and C_17_H_13_O_8_^−^, Table 1 [27]. Among those, peaks 11 and 12 were identified as the isomers 3′,5′-dimethoxymyricetin and 3′,4′-dimethoxymyricetin [27], respectively. In the same way, peak 26 was identified as the tetramethyl flavone 5,7-dihydroxy-3,8,3′,4′-tetramethoxyflavone (C_19_H_17_O_8_^−^) [15]. Peaks 16 and 20 with [M – H]^−^ ions at *m*/*z*: 359.07715 (Figure S1d) and 389.08780 (Figure S1g) were identified as 7,3′,5′-trimethoxymyricetin and 6-hydroxy-3,7,3′,5′-tetramethoxymyricetin [27] (C_18_H_15_O_8_^−^, C_19_H_17_O_9_^−^), respectively. 

#### 2.2.4. Flavanones

Peak 5 was identified as the flavanone eriodictyol (C_15_H_11_O_6_^−^), peak 4 as Hydroxy-hesperetin (C_16_H_13_O_7_^−^), peak 9 and 19 as the derivatives hydroxyeriodictyol (C_15_H_11_O_7_^−^) and methoxyeriodictyol (C_16_H_13_O_6_^−^). In the same manner, peak 15 was identified as the flavanone hesperetin (C_16_H_13_O_6_^−^), which was isolated and confirmed by spiking experiments, and peak 4 was identified as hydroxyhesperetin (C_16_H_13_O_7_).

#### 2.2.5. Prenylated Flavonoids

Several interesting isoprenylated derivatives of flavonoids were also detected. Peak 10 with a pseudomolecular anion at *m*/*z*: 413.12411 was identified as the dimethoxylated isoprenylated myricetin derivative: 8-isoprenyl-7,4′-dimethoxymyricetin (C_22_H_21_O_8_^−^, Figure S1c), while peak 13 as the trimethoxylated derivative: 8-isoprenyl-7,3′,4′-trimethoxymyricetin (C_23_H_23_O_8_^−^), Peak 41 was identified as 8-isoprenyl-7-methoxyquercetin (C_21_H_19_O_7_^−^) and peak 21 as 8-isoprenyl-7,4′-dimethoxykaempferol (C_22_H_21_O_6_^−^). Peak 30 was identified as the derivative of the latter, 3-acetyl-8-isoprenyl-7,5,4′-trimethoxykaempferol (C_25_H_25_O_7_^−^) and peak 35 as the acetylated one, 3-*O*-acetyl-8-isoprenyl-7,4′-dimethoxykaempferol (C_24_H_23_O_7_^−^). Peak 38 was identified as 8-isoprenyl-7,4′-dimethoxyapigenin (C_22_H_21_O_5_^−^, Figure S1m). 

#### 2.2.6. Kaurene Terpenoids

Peak 18 with a [M − H]^−^ ion at *m*/*z*: 437.21780 (Figure S1f) was identified as the kaurene diterpenoid Adenolin C (C_23_H_33_O_8_^−^) and peaks 23 and 42 as its derivatives, 11-acetoxy-11,12- dehydrated adenolin C (C_25_H_33_O_9_^−^) and 11-acetoxy-7-methoxyadenolin C (C_26_H_37_O_9_^−^), respectively. Peak 32 was identified as the 11,12-dehydrated derivative of Adenolin C (C_23_H_31_O_7_^−^, Figure S1k).

#### 2.2.7. Clerodane Terpenoids

Bacchalineol was identified with peak 29 (Table 2) while bacchalineol 18-*O*-malonic acid, bacchalineol 18-*O*-malonate methyl ester ([M – H]^−^ ion at *m*/*z*: 401.23328, C_24_H_33_O_5_^−^, Figure S1n,e, respectively), and bachalineol 18-*O*-malonate ethyl ester (Figure S1i) [25] were isolated and identified in the chromatograms with peaks 17, 25, and 28, identity confirmed with spiking experiments with authentic standards. Peak 34 with a [M – H]^−^ ion at *m*/*z*: 331.19141 was identified as the furanyl clerodane diterpene hawtriwaic acid (C_20_H_27_O_4_^−^) [30]. Other compounds detected were related to the antibacterial compound soligagoic acid A (C_20_H_29_O_2_^−^), [31,32]. Peak 33 with a [M – H]^−^ ion at *m*/*z*: 347.18637 was identified as 1,18-dihydroxysolidagoic acid (C_20_H_27_O_5_^−^), while peak 36 with a pseudomolecular ion at *m*/*z*: 359.22278 was identified as the hydroxyl-acetyl derivative of the alcohol, 19-hydroxy-solidagoiol A acetate (C_22_H_31_O_4_^−^, Figure S1l), and finally peak 31 as the *tri*-hydroxy-derivative: 1,2,19-trihydroxy-18-acetyl-solidagoiol A (C_22_H_31_O_6_^−^) [31,32]. Peak 39 with a [M – H]^−^ ion at *m*/*z* 401.23328 was identified as the related clerodane compound barticulidiol diacetate (C_24_H_33_O_5_^−^) [33], Peak 40 with an anion at *m*/*z*: 343.22784 was identified as the acetyl derivative of the alcohol, bacchalineol acetate (C_22_H_31_O_3_^−^, Figure S1n) [25,34]. 

#### 2.2.8. Tremetones 

Peak 27 with a [M – H]^−^ ion at *m*/*z*: 363.12385 was identified as *p*-coumaroyloxyltremetone, identity confirmed by spiking experiments with a standard sample, and peak 37 with a [M – H]^−^ ion at *m*/*z*: 347.12855 as a derivative of the latter, which was isolated and used as standard for spiking experiments (see experimental).

### 2.3. Gastroprotective Capacities of Isolated Compounds (***1**–**7***) from Parastrephia Quadrangularis

The results of the gastroprotective effects of compounds **1**–**7** in the HCl/EtOH-induced gastric lesion model are presented in Table 2. All compounds tested showed gastroprotective activity at a dose of 20 mg/kg (p.o) except compound **2** and **4**. The best effect was shown by compound **7** (76%) which was close to that observed with lansoprazole (72%). In addition, the protection displayed by compound **6** (41%) was nearly half of that showed by the positive control, showing that an addition of an OH group in the cinnamoyl moiety for compound **7** is key for the bioactivity. Among the most lipophilics compounds (**1**–**5**), the lowest gastroprotective activity was evidenced by compound **5** (bacchalineol 18-*O*-acetate, 19%), compound **1** (bacchalineol, 12%), and compound **3** (bacchalineol methyl malonate, 11%). Compound **2** (bacchalineol malonic acid) and **4** (bacchalineol 18-*O*-malonate ethyl ester) did not show any significant difference with the control group.

The gastroprotective activity of several terpenoids have been reported in the literature. Among them, plaunotol, ferruginol and their derivatives, mulinane diterpenoids, dehydroabietic acid derivatives, carnosol and carnosic acid derivatives, labdane diterpenoids, poligodial sesquiterpenoids, oleanolic acid and their derivatives, suaveolol diterpenoid, ent-beyerene derivatives, and lupeol [35,36,37,38,39,40]. The gastroprotective effects of these terpenoids reported in those studies were comparable with our results at the same oral dose. A high amount of compound **7** could explain in part for some of the putative medicinal properties assigned to this species. Further studies are necessary to explain the mechanism of action of compound **7**. 

## 3. Materials and Methods

### 3.1. Chemicals and Plant Material

UHPLC-MS Solvents, LC-MS formic acid, reagent grade lanzoprasole, formalin, ethanol, HCl, deuterated chloroform and deuterated methanol, and reagent grade chloroform were from Merck (Santiago, Chile). Ultrapure water was obtained from a Millipore water purification system (Milli-Q Merck Millipore, Santiago, Chile). UHPLC standards, (kaempferol, caffeic acid, isorhamnetin, hesperetin, eriodictyol, all standards with purity higher than 95% by HPLC) were purchased either from Sigma Aldrich (Saint Louis, Mo, USA), ChromaDex (Santa Ana, CA, USA), or Extrasynthèse (Genay, France). 

### 3.2. Plant Material

*Parastrephia quadrangularis* (Meyen), Cabrera aerial parts were collected in El Tatio, Atacama Desert, in November 2015 at 4000 m.a.s.l. and were identified by the botanist Alicia Marticorena from the University of Concepción, Chile. Voucher herbarium specimens are kept at the Natural Products Laboratory of the Universidad de Antofagasta under reference number: PQ20151115.

### 3.3. Extraction

Dried and chopped aerial parts of *P. quadrangularis* (500 g) collected in Northern Chile were extracted with absolute ethanol (1 L, per 3 times in the dark, 24 h each time) to obtain a medicinal tincture, at room temperature. The tincture was then concentrated in vacuum below 40 °C to yield 36 g of a dark gummy extract.

### 3.4. Isolation

Thirty-six grams of the crude ethanol extract (concentrated medicinal tincture) was submitted to flash permeation thorough Sephadex LH-20 (700 g) using methanol as eluent and three fractions (PQ-A to PQ-C) were collected after TLC analyses and clear spots (Kieselgel F254 plates, developed with Hexane: EtOAc 8:2 *v*/*v*, and spots visualized by spraying with vanillin:sulfuric acid 2% in ethanol and heating) . Fraction PQ-B (7 g) was submitted to open column chromatography (Silica gel 60, 500 g) using hexane ethyl acetate of increasing polarity and 7 fractions were collected (pq-a to pq-f) according to TLC profiles. Fractions pq-d and pq-f were pooled, (2 g) and submitted to a medium pressure column chromatography system composed of an 2.5 cm × 48 cm medium pressure column (Aceglass Inc., Vineland, NY, USA) packed with silicagel (Kieselgel 60 H, Merck, Darmstadt, Germany) using an isocratic solvent system of n-hexane-ethyl acetate (9.5:0.5 *v*:*v*) pumped with a medium pressure pump (FMI lab pump, Syosset, NY, USA) with a flow rate of 10 mL-minute. The collected fractions (75) were combined according to TLC analysis and 12 combined fractions (Pq-1 to Pq-12) were obtained. Fraction Pq-3 (376 mg) was re-chromatographed using the same chromatographic system and the known compounds: **1** (32 mg) and **2** (44 mg) were isolated which showed similar NMR spectra [25]. Fraction Pq-5 (543 mg) was rechromatographed on Sephadex L-H 20 to yield diterpenes **3** (35 mg), **4** (43 mg) and **5** (50 mg) whose NMR data corresponded to the data previously reported [25]. Fraction Pq-7 (612 mg) was rechromatographed on Sephadex L-H 20 to yield the new tremetone compound **6** (42 mg) plus the known tremetone **7** (55 mg) [26]. From fraction PQ-C (5 g), after several steps on Sephadex LH-20 permeation (500 g and 200 g, using as mobile phase HPLC grade methanol), the methylated flavones 5,7-dihydroxy-3,8,3′,4′-tetramethoxyflavone (13 mg) 3′,4′-dimethoxymyricetin (23 mg), 3,7,3′-trimethoxyquercetin (17 mg), plus hesperetin (12 mg), showing NMR data as previously reported [15,27,41] plus the coumarins umbelliferone (15 mg) and scopoletin (23 mg) [28], were isolated. 

### 3.5. UHPLC-PDA-MS Instrument

A Thermo Scientific Dionex Ultimate 3000 UHPLC system hyphenated with a Thermo Q exactive focus machine was used [24]. For the analysis, 5 mg of the extract were dissolved in 2 mL of methanol, filtered (thorough PTFE filter) and 10 µL were injected in the instrument, with all specifications set as previously reported [24].

### 3.6. LC Parameters and MS Parameters

Liquid chromatography was performed using an UHPLC C18 column (Accucore, 150 mm × 4.6 mm internal diameter, 2.5 μm particle size, Thermo Fisher Scientific, Bremen, Germany) operated at 25 °C. The detection wavelengths were 254, 280, 330 and 354 nm, and PDA was recorded from 200 to 800 nm for peak characterization. Mobile phases were 1% formic aqueous solution (A) and 1% formic acid in acetonitrile (B). The gradient program (time (min), % B) was: (0.00, 12); (5.00, 12); (10.00, 20); (15.00, 40); (20.00, 40); (25.00, 70); (35.00, 12) and 15 min for column equilibration before each injection. The flow rate was 1.00 mL min^−1^, and the injection volume was 10 µL. Standards and the resin extract dissolved in methanol were kept at 10 °C during storage in the autosampler. The HESI II and Orbitrap spectrometer parameters were optimized as previously reported [24,42].

### 3.7. Animals

Animals were acquired from the Chilean Institute of Health, Chile, Santiago. Swiss albino mice weighing 30 ± 3 g were fasted for 24 h before the experiments. The animals were fed on certified Champion diet with free access to water under standard conditions of 12 h dark-light period, 50% relative humidity and room temperature (22 °C). The protocols were approved by the Animal Use and Care Committee of the Universidad de Chile (07022010) following the recommendations of the Canadian Council on Animal Care as stated previously [40]. 

### 3.8. Gastroprotective Effects

The gastroprotective activity of the compounds **1**–**7** was performed in the HCl/EtOH-induced lesion model as described previously [19]. Briefly, mice were distributed into groups of seven animals each and fasted for 24 h with free access to water prior to the experiments. Fifty min after oral administration of the compounds (20 mg/kg), lansoprazole (20 mg/kg) or 12% Tween 80 (10 mL/kg), all groups were orally treated with 0.2 mL of a solution containing 0.3 M HCl/60% ethanol (HCl/EtOH) for gastric lesion induction. Animals were sacrificed 1 h after the administration of HCl/EtOH, and the stomachs were excised and inflated by injection of saline (1 mL). The ulcerated stomachs were fixed in 5% formalin for 30 min and opened along the greater curvature. Gastric damage visible to the naked eye was observed in the gastric mucosa as elongated black-red lines, parallel to the long axis of the stomach. The length (mm) of each lesion was measured, and the lesion index was expressed as the sum of the length of all lesions.

### 3.9. Statistical Analysis 

The statistical analysis was carried out using the originPro 9.1 software packages (Originlab Corporation, Northampton, MA, USA). The determination was repeated at least three times for each sample solution. Analysis of variance was performed using ANOVA. Significant differences between means were determined by Dunnet comparison test (*p* values < 0.05 were regarded as significant). 

## 4. Conclusions

The ethanol extract of an endemic species from the Atacama Desert showed several metabolites which were isolated using chromatography and detected using a hybrid UHPLC-PDA-OT-MS instrument. The isolated tremetones and clerodanes showed gastroprotective activity in a mouse model, evidenced by compound **7**, which showed better gastroprotective capacity than the control drug lansoprazole (76%). The hyphenated machine equipped with orbitrap-PDA detectors and high-resolution collision cell is an outstanding tool for accurate and fast metabolomics analysis of the Atacama Desert flora, and allowed for the first time the identification of several *ent*-clerodane and kaurene diterpenes. *P. Quadrangularis* is rich in phenolic compounds and terpenoids and thus can be useful for the preparation of nutritional supplements. This study might support in part the putative medicinal properties of the plant as a gastroprotective agent. 

## Figures and Tables

**Table 1:** UHPLC PDA and HR-MS analysis of *P. quadrangularis* ethanol extract.

Peak Number	UV Max (nm)	Tentative Identification	Elemental Composition [M – H]^−^	Retention Time (min)	Theoretical Mass (*m*/*z*)	Measured Mass (*m*/*z*)	MS^n^ Ions
1	238–310	Dicaffeoyl quinic acid	C_25_H_23_O_12_^−^	10.65	515.11938	515.11840	353.08774, chlorogenic acid, 191.05554 (quinic acid)
2	270–310	Euphorbetin	C_18_H_9_O_8_^−^	11.92	353.03046	353.02919	177.01871
3	310	Caffeic acid *	C_9_H_7_O_4_^−^	12.62	179.03498	179.03441	108.02070(C_6_H_8_O_2_^−^; –CH=CHCOO–)
4	288	Hydroxy-hesperetin	C_16_H_13_O_7_^−^	13.24	317.06668	317.06656	125.02354 (C_6_H_5_O_3_^−^); 207.06560 (C_11_H_11_O_4_^−^)
5	281	Hydroxyeriodictyol	C_15_H_11_O_7_^−^	11.92	303.05103	303.05090	125.02360 (C_6_H_5_O_3_^−^)
6	265–365	Kaempferol *	C_15_H_9_O_6_^−^	13.67	285.04046	285.04028	135.04431 (C_8_H_7_O_2_^−^)
7	254–365	Isorhamnetin *	C_16_H_11_O_7_^−^	14.23	315.05103	315.05090	300.05090 (C_15_H_8_O_7_^−^)
8	255–375	6-Hydroxytrimethoxymyricetin	C_18_H_15_O_9_^−^	15.55	375.07211	375.07202	315.01437 (C_15_H_7_O_8_^−^); 271.02448 (C_14_H_7_O_5_^−^)
9	287	Eriodictyol *	C_15_H_11_O_6_^−^	13.55	287.05611	287.05597	243.02946 (C_13_H_7_O_5_^−^)
10	255–373	8-Isoprenyl-7,4′-dimethoxymyricetin	C_22_H_21_O_8_^−^	15.97	413.12419	413.12411	145.02867 (C_9_H_5_O_2_^−^); 249.07637 (C_13_H_13_O_5_^−^)
11	255–375	3′,5′-Dimethoxymyricetin	C_17_H_13_O_8_^−^	14.36	345.06159	345.06152	315.01422 (C_15_H_7_O_8_^−^)
12	255–373	3′,4′-Dimethoxymyricetin *	C_18_H_15_O_8_^−^	16.86	345.06159	345.06149	285.04007 (C_15_H_9_O_6_^−^); 125.02357 (C_6_H_5_O_3_^−^);
13	255–373	8-Isoprenyl-7,3′,4′-trimethoxymyricetin	C_23_H_23_O_8_^−^	17.64	427.13984	427.13977	145.02869 (C_9_H_5_O_2_^−^); 263.09210 (C_14_H_15_O_5_^−^)
14	254–365	7,3′-Dimethoxyquercetin(7-*O*-methyl-isorhamnetin)	C_17_H_13_O_7_^−^	18.21	329.06668	329.06662	299.01953 (C_15_H_7_O_7_^−^)
15	287	Hesperetin *	C_16_H_13_O_6_^−^	18.42	301.07176	301.07159	135.04431 (C_8_H_7_O_2_^−^)
16	255–373	7,3′,5′-Trimethoxymyricetin	C_18_H_15_O_8_^−^	15.98	359.07724	359.07715	284.03229 (C_15_H_8_O_6_^−^)
17	205	18-*O*-Malonyl-Bacchalineol	C_23_H_31_O_5_^−^	19.86	387.21770	387.21771	-
18	203	Adenolin C	C_23_H_33_O_8_^−^	19.43	437.21809	437.21780	299.01953 (C_15_H_7_O_7_^−^)
19	285	Methoxyeriodictyol	C_16_H_13_O_6_^−^	19.68	301.07176	301.07166	135.04430 (C_8_H_7_O_2_^−^)
20	255–373	6-Hydroxy-3,7,3′,5′-tetramethoxymyricetin	C_19_H_17_O_9_^−^	19.87	389.08781	389.08780	359.04031 (C_17_H_11_O_9_^−^, -2CH_3_)
21	265–365	8-Isoprenyl-7,4′-dimethoxykaempferol	C_22_H_21_O_6_^−^	20.01	381.13436	381.13425	119.04949 (C_8_H_7_O^−^)
22	205	11-Acetoxy-11,12-dehydrated adenolin C	C_25_H_33_O_9_^−^	20.25	477.21301	477.21249	-
23	217	11-Acetoxy-7-methoxyadenolin C	C_26_H_37_O_9_^−^	20.27	493.24431	493.24384	-
24	254–354	3,7,3′-Trimethoxyquercetin(3,7-di-*O*-methyl-isorhamnetin) *	C_18_H_15_O_7_^−^	20.49	343.08233	343.08203	313.03491 (C_16_H_9_O_7_^−^)
25	217	Bacchalineol 18-*O*-malonate methyl ester	C_25_H_35_O_5_^−^	20.52	401.23340	401.23225	-
26	255–373	5,7-Dihydroxy-3,8,3′,4′-Tetramethoxyflavone	C_19_H_17_O_8_^−^	20.84	373.09277	373.09271	343.04556 (C_17_H_11_O_8_^-^)
27	254–355	*p*-Coumaroyloxytremetone	C_22_H_19_O_5_^−^	20.93	363.12380	363.12378	121.02878 (C_7_H_5_O_2_^−^)
28	207	Bacchalineol 18-*O*-malonate ethyl ester	C_25_H_35_O_5_^−^	21.05	415.24899	415.24790	
29	218	Bacchalineol	C_20_H_29_O_2_^−^	21.26	301.21730	301.21840	-
30	265–365	3-*O*-Acetyl-8-isoprenyl-7,5,4′-trimethoxykaempferol	C_25_H_25_O_7_^−^	21.75	437.16058	437.16046	119.04943 (C_8_H_7_O^−^); 163.03926 (C_9_H_7_O_3_^−^)
31	210	1,2,19-Trihydroxy-18-acetyl-solidagoiol A	C_22_H_31_O_6_^−^	21.54	391.21261	391.21252	287.20145 (C_19_H_27_O_2_^−^)
32	207	Adenolin C 11,12 dehydrated derivative	C_23_H_31_O_7_^−^	21.76	419.20753	419.20746	289.21698 (C_19_H_29_O_2_^−^)
33	207	1,18-Dihydroxysolidagoic acid	C_20_H_27_O_5_^−^	22.01	347.18640	347.18637	
34	207	Hawtriwaic acid	C_20_H_27_O_4_^−^	22.45	331.19148	331.19141	-
35	265–365	3-*O*-Acetyl-8-isoprenyl-7,4′-dimethoxykaempferol	C_24_H_23_O_7_^−^	22.58	423.14493	423.14487	119.04947 (C_8_H_7_O^−^); 163.03931 (C_9_H_7_O_3_^−^)
36	205	19-Hydroxy-solidagoiol A acetate	C_22_H_31_O_4_^−^	22.78	359.22278	359.22278	211.07591 (C_14_H_11_O_2_^−^)
37	-	*p*-Cinammoyloxytremetone	C_22_H_19_O_4_^−^	22.86	347.12888	347.12892	
38	232–272	8-Isoprenyl-7,4′-dimethoxyapigenin	C_22_H_21_O_5_^−^	23.66	365.13945	365.13947	119.04942 (C_8_H_7_O^−^); 201.09149 (C_13_H_13_O_2_^−^)
39	205	Barticulidiol diacetate	C_24_H_33_O_5_^−^	24.15	401.23335	401.23328	333.20688 (C_20_H_29_O_4_^−^)
40	205	Bacchalineol acetate	C_22_H_31_O_3_^−^	25.74	343.22787	343.22784	-
41	254–355	8-Iisoprenyl-7-methoxyquercetin	C_21_H_19_O_7_^−^	26.35	383,11363	383.11353	119.04935 (C_8_H_7_O^−^); 163.03922 (C_9_H_7_O_3_^−^)
42	270–310	Umbelliferone	C_9_H_6_O_3_^−^	26.43	162.03169	162.03124	
43	270–310	Scopoletin		26.78	192.04226	192.04220	

* Identification made using authentic standards.

**Table 2:** Gastroprotective effect of compounds isolated from *P. quadrangularis* at 20 mg/kg on HCl/EtOH-induced gastric lesions in mice.

Compound	*n*	Lesion Index (mm)	% Lesion Reduction
**1**	7	35.7 ± 4.6 **	12 *
**2**	7	39.0 ± 3.5 **	4
**3**	7	36.6 ± 1.5 **	11 *
**4**	7	46.6 ± 7.2 **	-
**5**	7	33.1 ± 2.0 **	19 *
**6**	7	23.9 ± 3.1 **	41 *
**7**	7	13.9 ± 2.2	76 *
Lansoprazole	7	11.3 ± 1.5	72 *
Control	7	40.6 ± 1.2 **	-

The results are expressed as mean ± sem * *p* < 0.01; significantly different compared with the control and ** *p* < 0.01 significantly different compared with lansoprazole (ANOVA followed by Dunnett’s test). *n* = number of mice.

## Supplementary Materials

The following are available online, NMR data and Full mass spectra and structure of several of the compounds identified by UHPLC-ESI-MS-MS.
